# Type 2 Diabetes in Taiwan: Unmasking Influential Factors Through Advanced Predictive Modeling

**DOI:** 10.1155/jdr/5531934

**Published:** 2025-05-27

**Authors:** Shih-Tsung Chang, Ying-Hsiang Chou, Oswald Ndi Nfor, Ji-Han Zhong, Chien-Ning Huang, Yung-Po Liaw

**Affiliations:** ^1^Institute of Medicine, Chung Shan Medical University, Taichung City, Taiwan; ^2^Department of Radiation Oncology, Chung Shan Medical University Hospital, Taichung City, Taiwan; ^3^School of Medicine, Chung Shan Medical University, Taichung City, Taiwan; ^4^School of Medical Imaging and Radiological Sciences, Chung Shan Medical University, Taichung City, Taiwan; ^5^Department of Public Health, Institute of Public Health, Chung Shan Medical University, Taichung City, Taiwan; ^6^Department of Internal Medicine, Chung Shan Medical University Hospital, Taichung City, Taiwan; ^7^Department of Medical Imaging and Radiological Sciences, Chung Shan Medical University, Taichung City, Taiwan

**Keywords:** predictive models, risk factors, Type 2 diabetes

## Abstract

**Background:** Type 2 diabetes (T2D) is influenced by lifestyle, genetics, and environmental conditions. By utilizing machine learning techniques, we can enhance the precision of T2D risk prediction by analyzing the complex interactions among these variables. This study was aimed at identifying and predicting key variables linked to T2D within the Taiwanese population.

**Methods:** The study included 3623 individuals with T2D and 14,492 without. Data on lifestyle and anthropometric measures were obtained from the Taiwan Biobank. Statistical analyses were performed using Base SAS software and SAS Viya.

**Results:** Traditional models identified body mass index (BMI) and waist–hip ratio (WHR) as significant risk factors for T2D, with odds ratios (OR) of 1.10 (95% confidence interval (CI) 1.09–1.12) and 1.10 (95% CI 1.09–1.11), respectively. These variables remained crucial in predictive models, with the WHR being the most influential. In the overall population, BMI's relative importance was 0.57, differing by gender (0.23 in men and 0.62 in women). While cigarette smoking and certain genetic variants (*CDKAL1*, *SLC30A8*, *CDKN2B*, *KCNQ1*, *HHEX*, *and TCF7L2*) were significant in traditional models, their importance decreased in predictive models.

**Conclusions:** Among various factors, the WHR emerged as the most critical attribute for T2D, underscoring the complexity of T2D etiology. Overall, the random forest and ensemble classifiers emerge as the most effective models, especially in mixed and female categories, highlighting their robustness in predictive performance.

## 1. Introduction

Type 2 diabetes (T2D) is a chronic metabolic disorder primarily characterized by inadequate insulin production or the body's ineffective use of insulin, leading to elevated blood glucose levels [1]. The global prevalence of T2D has been increasing, making it a significant public health challenge [2]. In Taiwan, diabetes is a leading cause of death, with nearly 10,000 deaths annually and approximately 2 million individuals affected, a number that grows by 25,000 new cases each year [3]. By 2030, it is expected to impact approximately 552 million individuals worldwide [4].

The World Health Organization reported a dramatic rise in diabetes prevalence, with the number of affected individuals increasing from 108 million in 1980 to 422 million in 2014. This increase has been more pronounced in low- and middle-income countries compared to high-income countries. Between 2000 and 2019, diabetes mortality rates by age rose by 3% [5].

The development of T2D is influenced by a combination of genetic, lifestyle, and environmental factors [5–9]. Key lifestyle risk factors include family history, obesity, poor diet, physical inactivity, and hypertension [5, 10–12]. Research has identified multiple genetic variations associated with T2D. While genetic variations significantly affect insulin secretion and glucose metabolism, maintaining a healthy lifestyle [5] can mitigate these risks [13–17]. Although extensive research has explored these elements individually, there remains a need for comprehensive studies that integrate diverse variables to enhance our understanding of T2D etiology, particularly within specific populations.

Machine learning models have emerged as powerful tools in disease risk prediction, especially for complex conditions like T2D. These models utilize various algorithms, such as support vector machines, random forests, and deep learning techniques, to analyze vast datasets encompassing genetic, lifestyle, and environmental variables. This approach helps identify patterns and interactions that traditional statistical methods may overlook. For instance, studies have shown that machine learning can improve the accuracy of cardiovascular disease risk predictions by integrating diverse data sources, including electronic health records and genomic information [18]. Additionally, the application of machine learning in predicting the onset of diseases like T2D has shown promise, as these models can adapt and improve over time with new data, leading to more personalized healthcare interventions [19]. Machine learning approaches offer alternative methods for data extraction, allowing for legitimate conclusions despite inherent pitfalls [20]. These methods are believed to provide reliable risk prediction [21]. The ability of machine learning to continuously learn and adapt from new data enhances its predictive accuracy, making it a valuable asset in public health and clinical settings. Furthermore, these models can facilitate early intervention strategies, enabling healthcare providers to target at-risk populations more effectively and improve health outcomes.

The Taiwanese population presents a unique demographic, including distinct genetic and lifestyle factors. Investigating the interplay between these lifestyle factors, genetic variants, and anthropometric measures is crucial for tailoring effective preventive strategies and interventions. Moreover, integrating advanced analytics tools, such as Base SAS software and SAS Viya, enables a sophisticated analysis of large-scale datasets, facilitating the identification of crucial predictors and refining predictive models. In this investigation, we employed this software to evaluate how genetic and lifestyle factors influence T2D.

## 2. Materials and Methods

### 2.1. Data Collection and Variable Definition

We integrated the Taiwan Biobank questionnaire data with the 17 single-nucleotide polymorphisms (SNPs) selected. T2D was defined using the following criteria: fasting glucose levels ≥ 126 mg/dL, HbA1c ≥ 6.5%, or self-reported physician-diagnosed diabetes. Additional variables included continuous measures: age, body mass index (BMI), and categorical variables: sex (female/male), cigarette smoking (yes/no), alcohol consumption (yes/no), exercise habits (yes/no), vegetarian diet (yes/no), and hypertension status (yes/no). Regular exercise was defined as “engaging in moderate to vigorous physical activity at least three times per week, with each session lasting at least 30 minutes.” Regular smokers were defined as individuals who had smoked cigarettes for at least 6 months and continued to smoke at the time of data collection. Drinkers were those who reported a consistent habit of consuming at least 150 mL of alcohol per week for 6 months or longer.

### 2.2. Sample Size and Propensity Score Matching

**Figure 1:**
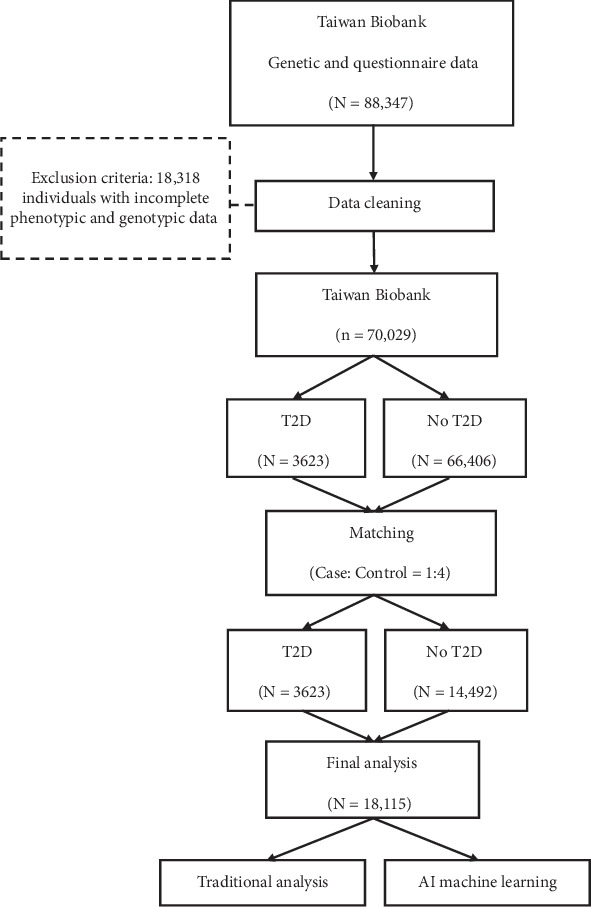
Flowchart of the study.

Individuals with missing data in either the questionnaire or genetic datasets (*n* = 18,318) were excluded, resulting in a total sample size reduction from 88,347 to 70,029 individuals. Among these, 544 were classified as T2D patients (cases), while 66,406 were non-T2D individuals (controls). We performed propensity score matching at a case-to-control ratio of 1:4, using sex and age as matching criteria. This procedure yielded a final sample of 18,115 individuals, comprising 3623 in the case group and 14,492 in the control group (Figure 1).

### 2.3. SNP Selection

A comprehensive literature review identified 17 SNPs associated with T2D: rs4402960, rs1470579, rs7756992, rs13266634, rs3802177, rs10811661, rs1111875, rs7923837, rs7903146, rs2237892, rs2283228, rs2237895, rs5219, rs9939609, rs11868035, rs1889018, and rs6502618. The selection process was guided by a thorough review of existing literature that has consistently identified these SNPs as significant contributors to T2D risk across various populations, including Asian cohorts. Notably, variants such as *TCF7L2*, *CDKAL1*, and *SLC30A8* have been implicated in the pathogenesis of T2D due to their roles in insulin secretion and glucose metabolism. This functional relevance supports their inclusion in our analysis, particularly as they have been validated in multiple studies involving diverse populations, thereby reinforcing their importance in understanding T2D risk. By including these SNPs, we aim to provide insights into the genetic factors that contribute to T2D risk in Taiwan while also acknowledging the necessity for further validation of these associations in local cohorts.

### 2.4. Statistical Analyses

Distributions were compared using *t*-tests (for continuous variables) and chi-square tests (for categorical variables). Logistic regression was used to assess SNP and lifestyle associations with T2D under additive, dominant, and recessive models, adjusting for gender, age, BMI, smoking, alcohol consumption, exercise, and waist–hip ratio. SAS 9.4 (SAS Institute, Cary, NC, United States) and PLINK 1.90b (Shaun Purcell & Christopher Chang, URL: http://www.cog-genomics.org/plink/1.9/) were utilized.

Machine learning analyses were conducted using SAS Viya 3.5 (SAS Institute Inc., Cary, NC, United States). The classifiers included random forests, logistic regression, ensembles, Bayesian networks, and LASSO (Version 3.5, SAS Institute Inc., Cary, NC, United States). The training, validation, and testing data were divided into a 60:30:10 ratio. Inputs consisted of 17 SNPs, sex, age, BMI, smoking, alcohol, exercise, and waist–hip ratio; the target variable was T2D status. Performance was assessed using the AUC, sensitivity, specificity, and accuracy; the champion model was selected based on Youden's *J* statistics.

## 3. Results

Table 1 summarizes the characteristics of the 14,492 controls and 3623 T2D cases. A higher proportion of males was found among individuals with diabetes (44.08%) compared to those without diabetes (29.57%). Additionally, participants with diabetes were older on average (58.47 ± 7.76 years) compared to those without diabetes (49.70 ± 10.76 years). The BMI was also higher in the diabetes group (26.22 ± 4.34) compared to the diabetes-free group (23.90 ± 3.68), indicating a potential association between higher BMI and diabetes. Genotype frequencies also differed significantly between cases and controls for most SNPs analyzed. A larger proportion of individuals with diabetes were smokers (26.97%) compared to those without diabetes (17.17%). Similarly, alcohol consumption was more common among those with diabetes (11.45%) than among those without (7.71%). A higher percentage of individuals with diabetes engaged in regular exercise (50.76%) compared to the diabetes-free group (39.81%). The WHR was notably higher among individuals with diabetes (92.30 ± 6.30) compared to those without diabetes (85.90 ± 6.68).

Logistic regression results are shown in Table 2, modeling SNPs under additive, dominant, and recessive inheritance along with adjustment for sex, age, BMI, smoking, alcohol, exercise, and WHR. Across models, age, BMI, and WHR were consistently associated with increased T2D risk. In contrast, sex, alcohol drinking, and regular exercise did not show significant associations with T2D in any of the models. Several SNPs also exhibited significant associations across different models: rs7756992 (*CDKAL1*), rs7903146 (*TCF7L2*), rs2237895 (*KCNQ1*), rs3802177 (*SLC30A8*), rs10811661 (*CDKN2B*), rs1111875 (*HHEX*), rs7923837 (*HHEX*), rs2237892 (*KCNQ1*), rs5219 (*KCNJ11*), and rs11868035 (SREBF1).

**Figure 2:**
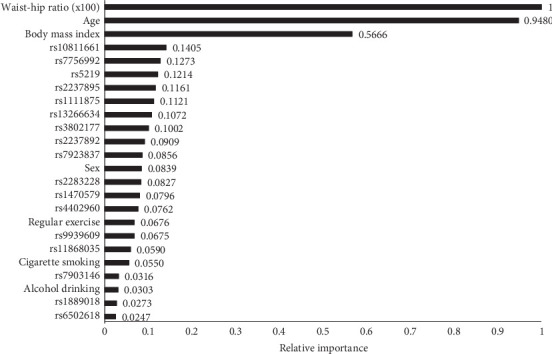
The relative importance of the selected T2D attributes identified by the champion model (random forest). The WHR was the most important attribute.

In the variable importance models (Figure 2), the most important attribute for T2D was consistently WHR, followed by age and BMI. Among the lifestyle factors and genetic variants tested, WHR emerged as the most important risk factor for T2D. Machine learning analyses using SAS Viya (Table 3) incorporated SNP genotypes, sex, age, BMI, smoking, alcohol drinking, exercise, and WHR as inputs, with T2D status as the target.

**Figure 3:**
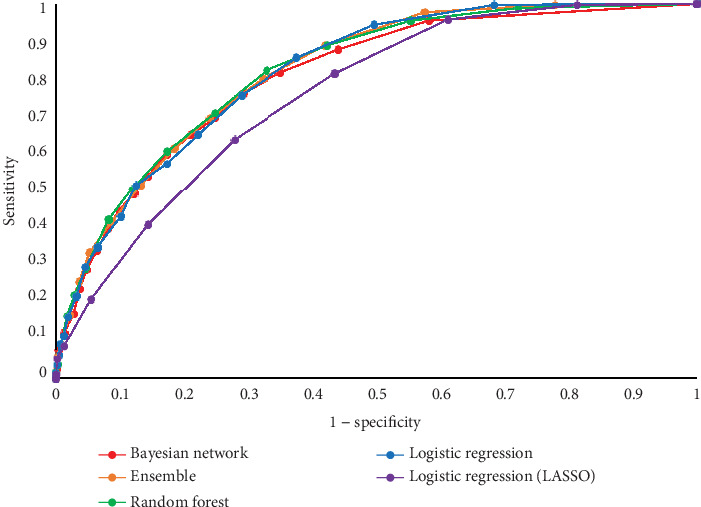
The receiver operating characteristic–area under the curve (ROC-AUC).

Top-performing (champion) models were random forests (overall and females) and ensembles (males). In the overall comparison, the ensemble classifier stood out with the highest AUC of 0.8211 and an accuracy of 0.7024, while the random forest led in sensitivity at 0.8232 and specificity at 0.6715 (Table 3 and Figure 3). In men, the ensemble classifier again excelled, achieving an AUC of 0.7824 and an accuracy of 0.6978. In females, the random forest classifier took the lead with an AUC of 0.8066 and an accuracy of 0.7002, demonstrating strong sensitivity at 0.8128. Throughout the analysis, LASSO logistic regression consistently ranked lower in performance across various metrics for both sexes.

## 4. Discussion

Our findings underscore the multifactorial nature of T2D risk within the Taiwanese population and highlight the value of leveraging advanced analytics. The machine learning models identified WHR as the paramount predictor of T2D, surpassing traditional attributes like age and BMI. While variables like smoking and certain genetic variants showed significance in logistic regression models, their relative importance diminished in the predictive algorithms, emphasizing the need for a holistic risk assessment approach.

The primacy of WHR over BMI and other anthropometrics in predicting T2D risk aligns with previous reports from Nepal [22]. In their case–control study, Radzevičienė et al. found that high BMI, waist circumference, and WHR were significant risk factors for T2D in women [23]. Research involving middle-aged men has similarly underscored the predictive value of central obesity measures in assessing the risk for T2D and cardiovascular diseases [24]. Age is also an established risk factor, potentially linked to metabolic dysregulation with aging [25]. Previous findings by Cheng and colleagues corroborate the waist–hip ratio as the superior anthropometric index for T2D prediction [26].

In the traditional logistic models, multiple SNPs (e.g., rs7756992, rs3802177, rs10811661, rs1111875, rs2237895, and rs5219) along with age, BMI, smoking, and WHR exhibited statistically significant associations with T2D, consistent with prior evidence in Asian populations [15, 27, 28]. However, machine learning analyses revealed a shift in relative importance, identifying WHR, age, and BMI as the primary predictors across both sexes, while the contributions of SNPs appeared more modest. Notable exceptions included rs10811661 and rs7756992, which maintained high rankings, especially in women and the overall population.

We employed multiple metrics to comprehensively evaluate model performance, including accuracy, precision, recall, *F*1-score, and AUC. The observed differences in model performance, particularly the lower accuracy of LASSO compared to other machine learning models, can be attributed to the inherent characteristics of the models, the nature of the data, and the complexity of the relationships being modeled. Despite these, it is essential to consider other metrics that may provide a more nuanced view of its predictive capabilities. For instance, LASSO may still yield valuable insights regarding feature importance, which can be critical for understanding the underlying factors contributing to T2D or other conditions. Notably, algorithms such as random forests and gradient boosting tend to capture intricate patterns more effectively by leveraging nonlinear decision boundaries and feature interactions. These models are generally more flexible and adaptive, which likely contributed to their superior predictive performance in our study.

The findings of our study underscore the importance of WHR as a significant predictor of T2D risk. Given its simplicity and cost-effectiveness, WHR screening could be integrated into routine clinical assessments. For instance, healthcare providers could incorporate WHR measurements into annual health check-ups, particularly for populations at higher risk of diabetes, such as those with a family history of the disease or those exhibiting other metabolic syndrome components [29, 30]. The evidence supporting WHR as a critical metric for assessing diabetes risk can inform public health policy in Taiwan and other regions facing similar health challenges. Policymakers could consider developing guidelines that recommend WHR screening as part of national diabetes prevention strategies. This could involve collaboration with various stakeholders, including healthcare providers, community organizations, and government agencies, to create a standardized approach to measure and report WHR. Similar to organized colorectal cancer screening programs that have demonstrated significant public health impact through increased screening rates, WHR screening could be promoted through community health fairs, school health programs, and workplace wellness initiatives [31].

Among the 17 SNPs included in our analysis model, *CDKAL* rs7756992, *SLC30A8* rs3802177, *CDKN2B* rs10811661, and KCNQ1 rs2237895 showed strong associations with T2D across multiple models, consistent with previous findings [15, 32]. This is consistent with findings from a replication study in Japan [15]. SLC30A8 rs3802177 and *CDKN2B* rs10811661 indicated potential protective effects in certain models contrary to previous findings in Asia and Europe [15, 27, 33]. The SNP *TCF7L2* rs7903146 showed a significant risk in additive and dominant models but not in the recessive model, reflecting variability in genetic influence. The remaining SNPs showed no significant associations with T2D. Notably, the overall SNPs were selected for analysis based on their previous associations with T2D in different ethnicities. Importantly, while these variants have been linked to T2D in various studies, their effect size and the degree of association can vary significantly between populations.

One of the primary limitations is the generalizability of our findings to non-Asian populations. The genetic and environmental factors influencing T2D can differ significantly across ethnic groups. For instance, while certain SNPs such as *TCF7L2* and *SLC30A8* have been associated with T2D in various populations, their effect sizes and associations may vary. This variability suggests that the risk factors identified in our study may not be directly applicable to populations outside of Taiwan, highlighting the need for caution when extrapolating our results to broader contexts. Next, the reliance on self-reported data for T2D status may have introduced reporting bias, as individuals may have misclassified their health status or failed to report their condition accurately. To minimize this, we also used fasting glucose levels ≥ 126 mg/dL and HbA1c ≥ 6.5% to define diabetes. Additionally, the cross-sectional design of our study limits our ability to infer causality between the identified risk factors and the development of T2D. Moreover, data on the intensity of certain behaviors—such as the number of cigarettes smoked per day, the precise volume of alcohol consumed, and the exact duration of physical activity—was not available in the dataset we used. Finally, the absence of longitudinal data may hinder the robustness of our findings and their implications for public health interventions aimed at mitigating T2D risk. We believe that future research incorporating longitudinal data would significantly enhance the validity of predictive models and their practical applications in public health settings.

## 5. Conclusions

Relative to factors such as BMI, smoking, alcohol consumption, and genetic variants, WHR emerged as the most critical predictor for T2D risk in this Taiwanese cohort, underscoring its pivotal role in diabetes risk assessment. The superior performance of the random forest and ensemble classifiers, particularly in mixed and female categories, highlights their robustness in predictive performance and underscores the importance of advanced analytical methods in T2D risk prediction. These findings provide valuable insights into the complex etiology of T2D, emphasizing the need for an integrative approach that combines genetic and advanced analytical methods to enhance disease risk prediction.

## Figures and Tables

**Table 1:** Descriptive characteristics of the study participants.

	**Diabetes-free (** **n** = 14,492**)**	**Diabetes (** **n** = 3623**)**	**p** ** value**
Sex			< 0.001
Female	10,206 (70.43%)	2026 (55.92%)	
Male	4286 (29.57%)	1597 (44.08%)	
Age	49.70 ± 10.76	58.47 ± 7.76	< 0.001
Body mass index	23.90 ± 3.68	26.22 ± 4.34	< 0.001
Cigarette smoking			< 0.001
No	12,003 (82.83%)	2646 (73.03%)	
Yes	2489 (17.17%)	977 (26.97%)	
Alcohol consumption			< 0.001
No	13,375 (92.29%)	3208 (88.55%)	
Yes	1117 (7.71%)	415 (11.45%)	
Regular exercise			< 0.001
No	8723 (60.19%)	1784 (49.24%)	
Yes	5769 (39.81%)	1839 (50.76%)	
Waist–hip ratio (×100)	85.90 ± 6.68	92.30 ± 6.30	< 0.001
rs4402960 (IGF2BP2)			< 0.001
GG	8450 (58.31%)	1952 (53.88%)	
GT	5208 (35.94%)	1461 (40.33%)	
TT	834 (5.75%)	210 (5.80%)	
rs1470579 (CDKAL1)			< 0.001
AA	8275 (57.10%)	1898 (52.39%)	
AC	5314 (36.67%)	1499 (41.37%)	
CC	903 (6.23%)	226 (6.24%)	
rs7756992 (CDKAL1)			< 0.001
AA	4338 (29.93%)	951 (26.25%)	
AG	7222 (49.83%)	1793 (49.49%)	
GG	2932 (20.23%)	879 (24.26%)	
rs13266634 (SLC30A8)			< 0.001
TT	4406 (30.40%)	1006 (27.77%)	
TC	6697 (46.21%)	1649 (45.51%)	
CC	3389 (23.39%)	968 (26.72)	
rs3802177 (SLC30A8)			< 0.001
GG	3741 (25.81%)	1088 (30.03%)	
GA	7502 (51.77%)	1805 (49.82%)	
AA	3249 (22.42%)	730 (20.15%)	
rs10811661 (CDKN2B)			< 0.001
TT	4847 (33.45%)	1413 (39.00%)	
TC	7124 (49.16%)	1713 (47.28%)	
CC	2521 (17.40%)	497 (13.72%)	
rs1111875 (HHEX)			< 0.001
CC	7110 (49.06%)	1635 (45.13%)	
CG	6101 (42.10%)	1614 (44.55%)	
GG	1281 (8.84%)	374 (10.32%)	
rs7923837 (HHEX)			0.007
AA	9430 (65.07%)	2280 (62.93%)	
AG	4548 (31.38%)	1181 (32.60%)	
GG	514 (3.55%)	162 (4.47%)	
rs7903146 (TCF7L2)			0.002
CC	13,840 (95.50%)	3410 (94.12%)	
CT	643 (4.44%)	210 (5.80%)	
TT	9 (0.06%)	3 (0.08%)	
rs2237892 (KCNQ1)			< 0.001
CC	6584 (45.43%)	1879 (51.86%)	
CT	6370 (43.96%)	1463 (40.38%)	
TT	1538 (10.61%)	281 (7.76%)	
rs2283228 (KCNQ1)			< 0.001
AA	5899 (40.71%)	1691 (46.67%)	
AC	6708 (46.29%)	1568 (43.28%)	
CC	1885 (13.01%)	364 (10.05%)	
rs2237895 (KCNQ1)			< 0.001
AA	6113 (42.18%)	1383 (38.17%)	
AC	6634 (45.78%)	1684 (46.48%)	
CC	1745 (12.04%)	556 (15.35%)	
rs5219 (KCNJ11)			0.046
CC	5672 (39.14%)	1337 (36.90%)	
CT	6813 (47.01%)	1760 (48.58%)	
TT	2007 (13.85%)	526 (14.52%)	
rs9939609 (FTO)			0.048
TT	11,214 (77.38%)	2747 (75.82%)	
TA	3061 (21.12%)	806 (22.25%)	
AA	217 (1.50%)	70 (1.93%)	
rs11868035 (SREBF1)			0.384
AA	10,871 (75.01%)	2714 (74.91%)	
AG	3381 (23.33%)	837 (23.10%)	
GG	240 (1.66%)	72 (1.99%)	
rs1889018 (SREBF1)			0.037
GG	12,890 (88.95%)	3232 (89.21%)	
GA	1560 (10.76%)	389 (10.74%)	
AA	42 (0.29%)	2 (0.06%)	
rs6502618 (SREBF1)			0.104
AA	12,883 (88.90%)	3222 (88.93%)	
AG	1591 (10.98%)	401 (11.07%)	
GG	18 (0.12%)	0 (0.00%)	

*Note:* Continuous variables are presented as mean ± standard deviations and categorical variables as *n* (%).

**Table 2:** Association of attributes with T2D based on the additive, dominant, and recessive models.

**Variables**	**M/m alleles**	**Additive model**		**Dominant model**		**Recessive model**	
		**aOR (95% CI)**	**p** ** value**	**aOR (95% CI)**	**p** ** value**	**aOR (95% CI)**	**p** ** value**
Gender (ref: Female)							
Male		1.02 (0.91–1.13)	0.788	1.01 (0.91–1.13)	0.804	1.02 (0.92–1.14)	0.679
Age		1.09 (1.08–1.10)	< 0.001	1.09 (1.08–1.10)	< 0.001	1.09 (1.08–1.09)	< 0.001
Body mass index		1.10 (1.09–1.12)	< 0.001	1.10 (1.09–1.12)	< 0.001	1.10 (1.09–1.12)	< 0.001
Cigarette smoking (ref: No)							
Yes		1.23 (1.09–1.38)	0.001	1.22 (1.08–1.38)	0.001	1.21 (1.07–1.37)	0.002
Alcohol consumption (ref: No)							
Yes		0.90 (0.78–1.05)	0.185	0.90 (0.78–1.05)	0.178	0.91 (0.79–1.06)	0.219
Regular exercise (ref: No)							
Yes		1.01 (0.93–1.10)	0.790	1.01 (0.93–1.11)	0.763	1.01 (0.93–1.11)	0.761
Waist–hip ratio (×100)		1.10 (1.09–1.11)	< 0.001	1.10 (1.09–1.11)	< 0.001	1.10 (1.09–1.11)	< 0.001
rs4402960 (IGF2BP2)	G/T	1.01 (0.78–1.31)	0.944	0.98 (0.73–1.31)	0.886	1.04 (0.60–1.79)	0.897
rs1470579 (CDKAL1)	A/C	1.14 (0.89–1.48)	0.301	1.25 (0.94–1.68)	0.127	1.01 (0.59–1.71)	0.974
rs7756992 (CDKAL1)	A/G	1.20 (1.13–1.27)	< 0.001	1.21 (1.10–1.33)	< 0.001	1.35 (1.23–1.49)	< 0.001
rs13266634 (SLC30A8)	T/C	0.94 (0.83–1.07)	0.362	1.07 (0.97–1.18)	0.203	1.16 (1.05–1.29)	0.003
rs3802177 (SLC30A8)	G/A	0.81 (0.70–0.92)	0.002	0.82 (0.74–0.90)	< 0.001	0.87 (0.78–0.97)	0.010
rs10811661 (CDKN2B)	T/C	0.79 (0.74–0.84)	< 0.001	0.74 (0.68–0.81)	< 0.001	0.71 (0.63–0.80)	< 0.001
rs1111875 (HHEX)	C/G	1.12 (1.04–1.20)	0.002	1.15 (1.05–1.26)	0.003	1.17 (1.01–1.36)	0.034
rs7923837 (HHEX)	A/G	1.09 (1.00–1.18)	0.053	1.07 (0.97–1.18)	0.162	1.33 (1.07–1.66)	0.010
rs7903146 (TCF7L2)	C/T	1.44 (1.20–1.72)	< 0.001	1.47 (1.22–1.76)	< 0.001	0.91 (0.18–4.52)	0.909
rs2237892 (KCNQ1)	C/T	0.83 (0.71–0.97)	0.021	0.76 (0.63–0.91)	0.004	0.80 (0.60–1.06)	0.119
rs2283228 (KCNQ1)	A/C	0.98 (0.84–1.13)	0.772	1.00 (0.83–1.20)	0.991	0.89 (0.69–1.16)	0.388
rs2237895 (KCNQ1)	A/C	1.11 (1.04–1.19)	0.003	1.11 (1.02–1.22)	0.022	1.40 (1.24–1.58)	< 0.001
rs5219 (KCNJ11)	C/T	1.09 (1.03–1.16)	0.006	1.11 (1.01–1.21)	0.022	1.14 (1.01–1.28)	0.030
rs9939609 (FTO)	T/A	1.07 (0.98–1.17)	0.138	1.06 (0.96–1.17)	0.241	1.30 (0.95–1.78)	0.100
rs11868035 (SREBF1)	A/G	1.07 (0.96–1.19)	0.243	1.02 (0.91–1.15)	0.739	1.55 (1.13–2.12)	0.006
rs1889018 (SREBF1)	G/A	0.71 (0.47–1.06)	0.090	0.75 (0.49–1.14)	0.178	0.26 (0.05–1.25)	0.092
rs6502618 (SREBF1)	A/G	1.30 (0.87–1.93)	0.197	1.30 (0.86–1.97)	0.219	< 0.01 (< 0.01–> 999.99)	0.954

*Note:* M/m alleles = major/minor alleles, ref = reference.

Abbreviation: aOR = adjusted odds ratio.

**Table 3:** Predictive performance on the testing set using SAS Viya.

	**AUC**	**Sensitivity**	**Specificity**	**Accuracy**	**F**1
Mixed					
Random forest	0.8208	0.8232	0.6715	0.7018	0.5246
Logistic regression	0.8191	0.8564	0.6239	0.6703	0.5094
Ensemble	0.8211	0.8011	0.6777	0.7024	0.5183
Bayesian network	0.8074	0.7624	0.7081	0.7189	0.5203
Logistic regression (LASSO)	0.7591	0.8149	0.5645	0.6146	0.4581
Male					
Ensemble	0.7824	0.7563	0.6760	0.6978	0.5762
Logistic regression	0.7731	0.7000	0.7133	0.7097	0.5671
Bayesian network	0.7680	0.7188	0.6853	0.6944	0.5610
Random forest	0.7669	0.7125	0.6876	0.6944	0.5588
Logistic regression (LASSO)	0.6804	0.8313	0.4848	0.5789	0.5175
Female					
Random forest	0.8066	0.8128	0.6778	0.7002	0.4735
Ensemble	0.8145	0.7931	0.6797	0.6985	0.4660
Logistic regression	0.8090	0.7488	0.7238	0.7279	0.4772
Bayesian network	0.7994	0.7389	0.7111	0.7157	0.4630
Logistic regression (LASSO)	0.7568	0.8768	0.5113	0.5719	0.4045

Abbreviations: AUC = area under the curve, LASSO = least absolute shrinkage and selection operator.

## Data Availability

Data are available from the authors upon reasonable request and with permission of the Taiwan Biobank.
